# Supplementary material for: Nanoscale Zero-Valent Iron Modified by Bentonite with Enhanced Cr(VI) Removal Efficiency, Improved Mobility, and Reduced Toxicity
Source: Nanomaterials (Basel). 2021 Sep 30;11(10):2580. doi: 10.3390/nano11102580 (PMC8537176; doi:10.3390/nano11102580)
Supplement: Supplementary file 1 [file nanomaterials-11-02580-s001.zip › nanomaterials-1372858.pdf]

Nanoscale Zero-Valent Iron Modified by Bentonite with Enhanced Cr(VI) Removal Efficiency, Improved Mobility, and Reduced Toxicity

Jien Ye [1,2], Yating Luo [1,2], Jiacong Sun [1,2] and Jiyan Shi [1,2,*]

[1] Department of Environmental Engineering, College of Environmental and Resource Sciences, Zhejiang University, Hangzhou 310058, China; yejien@zju.edu.cn (J.Y.); luoyating@zju.edu.cn (Y.L.); wssjc111@126.com (J.S.)
[2] MOE Key Laboratory of Environment Remediation and Ecological Health, College of Environmental & Resource Science, Zhejiang University, Hangzhou 310058, China
[*] Correspondence: shijiyan@zju.edu.cn; Tel.: +86-571-8898-2019

Contents:

Table S1 The particle-size class of test soils.

Table S2 The hydrodynamic diameter and zeta potential of quartz sand, bentonite and iron-based NPs.

Table S3 Pearson correlation analysis between mobility (breakthrough plateaus) and soil texture.

Table S4 The translocation factors(TF) of iron-based NPs and Fe-EDTA.

Figure S1 TEM and EDX images of nZVI and B-nZVI before and after the reaction.

Figure S2 The adsorption-desorption isotherms of nZVI and B-nZVI.

Figure S3 XRD patterns of nZVI and B-nZVI.

Figure S4 The removal of Cr(VI) by 1 g L^{-1} bentonite under different initial pH.

Figure S5 Fe 2p XPS spectra of reacted nZVI and B-nZVI.

Figure S6 The dry weight of ryegrass shoots (a) and roots (b). Error bars indicate the standard deviation of the mean (n = 3). Lowercase letters represent significant differences among the different treatments ($p < 0.05$).

Figure S7 Total Fe contents in the shoots and roots of perennial ryegrass. Error bars indicate the standard deviation of the mean (n = 3). Different letters represent significant differences among the different treatments ($p < 0.05$).

Table S1. The particle-size class of test soils.

Soil samples	Clay（%）	Silt（%）	Sand（%）	Soil type
S1	14.5	49.6	35.9	loam
S2	6.0	53.5	40.5	silt loam
S3	28.7	61.3	10.0	silt clay loam

Table S2. The hydrodynamic diameter and zeta potential of quartz sand, bentonite and iron-based NPs.

Sample	Hydrodynamic diameter (nm)	Zeta potential (mV)
Quartz sand	-	−40.31 ± 0.77
Bentonite	1325.7 ± 131.8	−36.20 ± 1.01
nZVI	1573.1 ± 272.9	5.07 ± 0.88
B-nZVI	1398.4 ± 96.1	−7.76 ± 1.32

Table S3. Pearson correlation analysis between mobility (breakthrough plateaus) and soil texture.

Soil texture	clay	silt	sand
**nZVI**	−0.873**	−0.332	0.732
**B-nZVI**	−0.994**	−0.762*	0.972*

*, correlation is significant at the 0.05 level ($p < 0.05$). **, correlation is significant at the 0.01 level ($p < 0.01$).

Table S4. The translocation factors(TF) of iron-based NPs and Fe-EDTA.

Treatment	TF
CK	0.524 ± 0.100
nZVI-50	0.170 ± 0.017
nZVI-200	0.191 ± 0.009
nZVI-500	0.183 ± 0.018
B-nZVI-50	0.399 ± 0.102
B-nZVI-200	0.206 ± 0.075
B-nZVI-500	0.176 ± 0.020
Fe-EDTA-50	0.500 ± 0.034
Fe-EDTA-200	0.645 ± 0.020
Fe-EDTA-500	0.695 ± z0.011

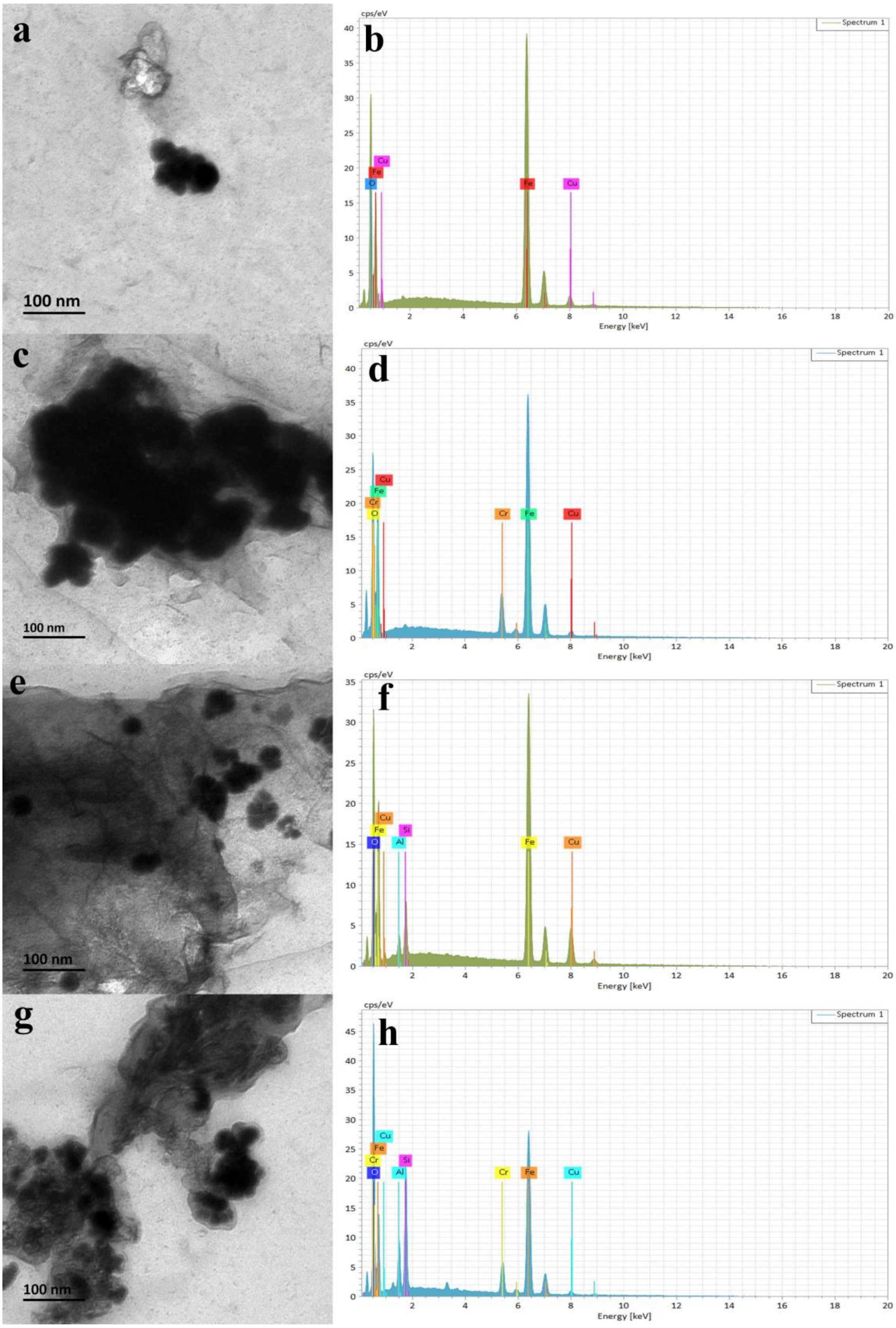

Figure S1. TEM and EDX images of nZVI and B-nZVI before and after the reaction. (nZVI before (**a**,**b**) and after (**c**,**d**) reaction; B-nZVI before (**e**,**f**) and after (**g**,**h**) after rection. The peak of copper is due to the use of copper foil as substrate).

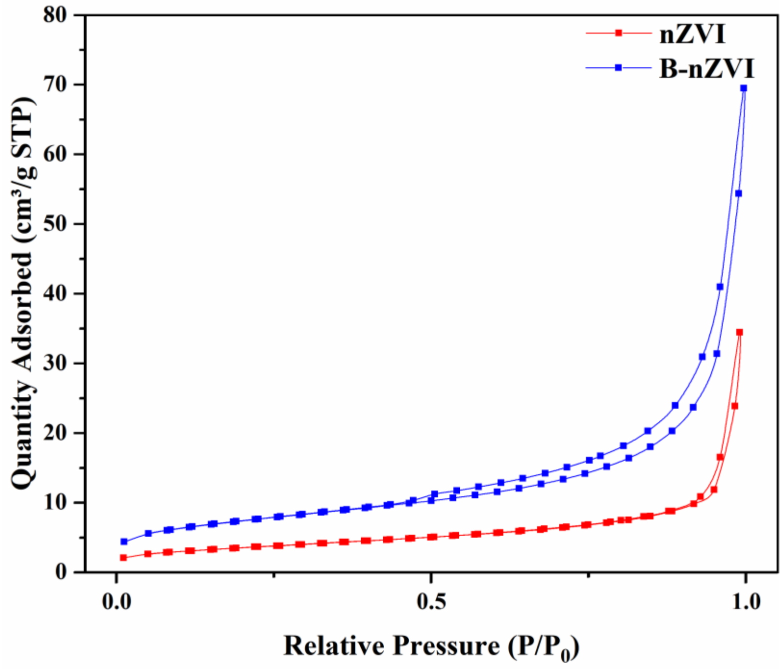

Figure S2. The adsorption-desorption isotherms of nZVI and B-nZVI.

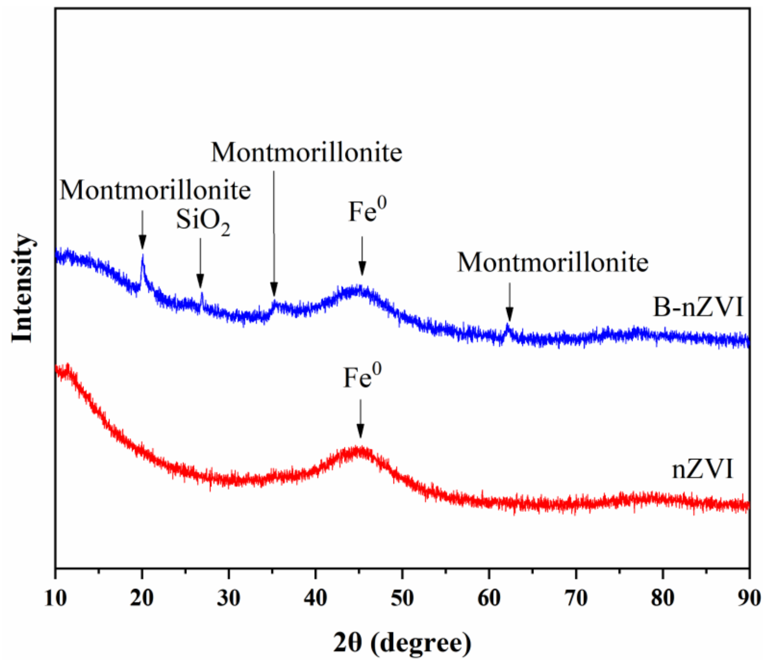

Figure S3. XRD patterns of nZVI and B-nZVI.

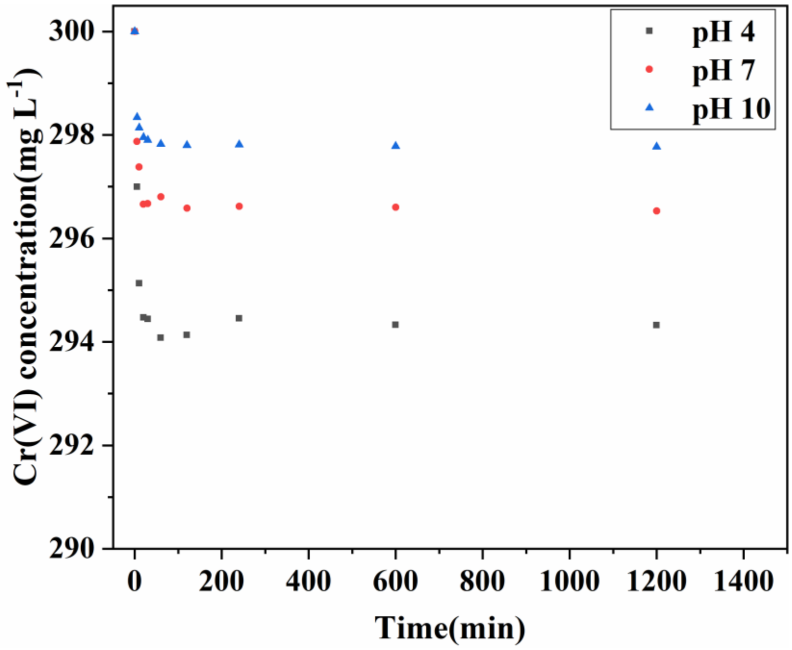

Figure S4. The removal of Cr(VI) by 1 g L^{-1} bentonite under different initial pH.

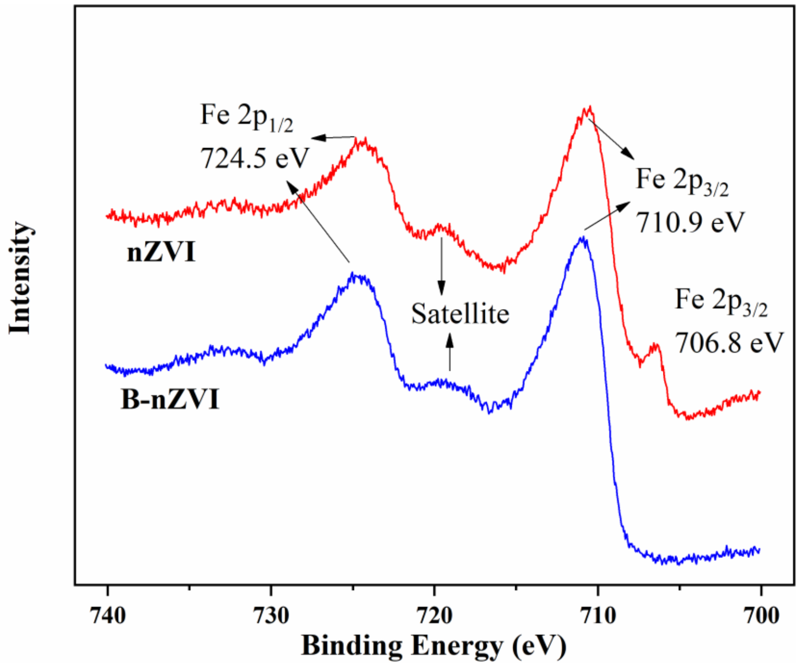

Figure S5. Fe 2p XPS spectra of reacted nZVI and B-nZVI.

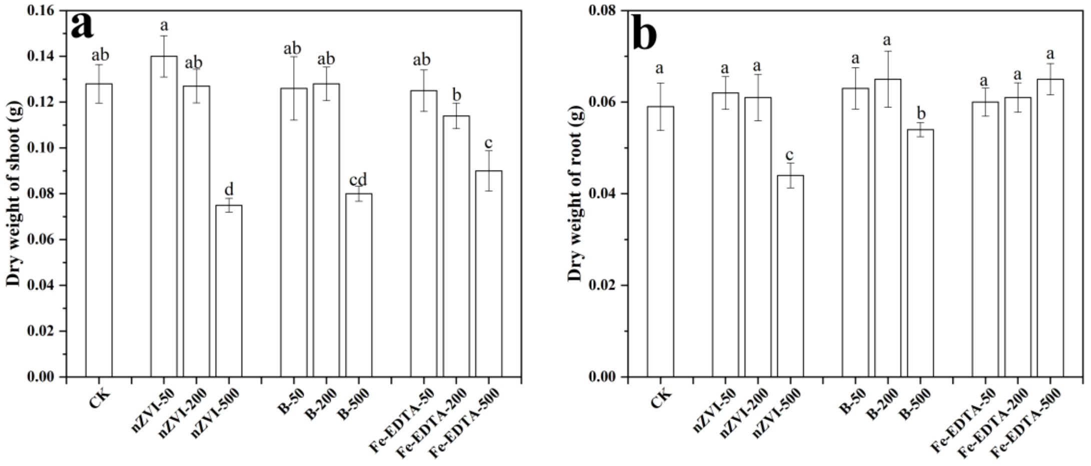

Figure S6. The dry weight of ryegrass shoots (**a**) and roots (**b**). Error bars indicate the standard deviation of the mean (n = 3). Lowercase letters represent significant differences among the different treatments (p < 0.05).

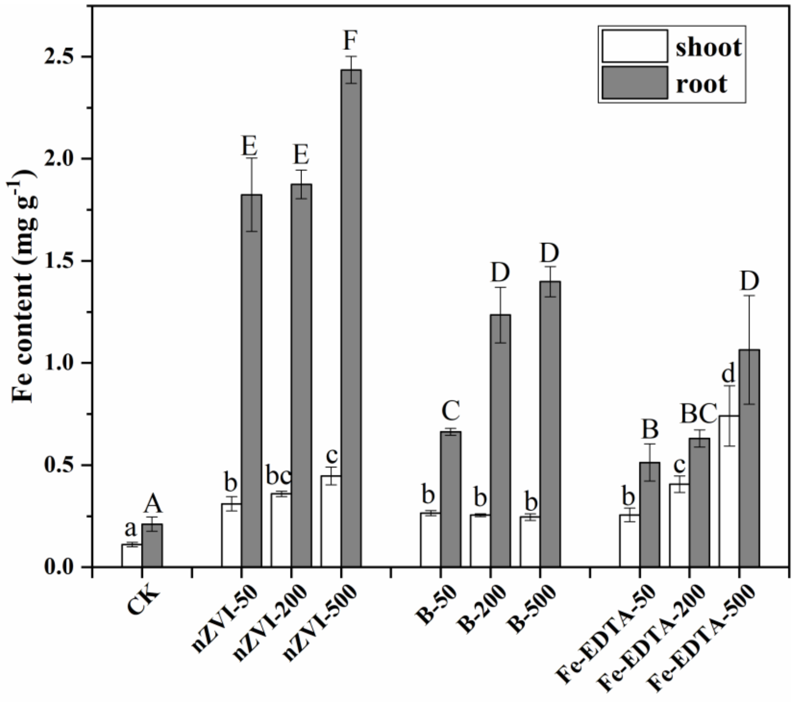

Figure S7. Total Fe contents in the shoots and roots of perennial ryegrass. Error bars indicate the standard deviation of the mean (n = 3). Different letters represent significant differences among the different treatments (p < 0.05).
